# Editorial: Focus on Exercise Physiology and Sports Performance: 2nd Edition

**DOI:** 10.3390/life15111671

**Published:** 2025-10-27

**Authors:** Laikang Yu

**Affiliations:** 1 Beijing Key Laboratory of Sports Performance and Skill Assessment, Beijing Sport University, Beijing 10084, China; yulaikang@126.com; 2 Department of Strength and Conditioning Assessment and Monitoring, Beijing Sport University, Beijing 10084, China

## 1. Introduction

Exercise physiology has developed into a dynamic scientific discipline that lies at the crossroads of biological science, medicine, and performance enhancement. It focuses on the physiological mechanisms that are activated during exercise and examines how these mechanisms can be applied to enhance health [1,2], prevent disease [3,4], and improve sports performance [5,6]. In recent decades, the field has evolved from traditional investigations centered on cardiovascular, respiratory, and muscular function to a broader exploration of molecular signaling [7], neuromuscular regulation [8], neuroplasticity [9], and exercise metabolism [10]. This expansion marks a transition from descriptive studies toward an integrative understanding of how different physiological systems interact dynamically to sustain and optimize performance.

A growing body of empirical evidence shows that exercise serves as a potent physiological stimulus, capable of eliciting both acute and long-term adaptations across multiple organ systems [11,12,13]. These adaptations include enhanced mitochondrial function, improved cardiovascular dynamics, remodeling of skeletal muscle fibers, and more efficient neuromuscular coordination. Such responses not only serve as the foundation for athletic success but also deliver substantial benefits for metabolic health, cognitive function, and overall longevity. Consequently, exercise physiology increasingly positions itself as a bridge between performance optimization and preventive medicine, emphasizing exercise’s dual role in supporting elite sports performance and promoting population-wide health [14,15,16].

At the same time, the study of sports performance has become more interdisciplinary, combining physiological principles with insights from biomechanics, psychology, nutrition, and data science. Researchers no longer limit their focus to answering how much exercise impacts performance; instead, they seek to unravel how and why specific training stimuli elicit individualized adaptive responses [17,18]. This paradigm shift has been driven largely by technological innovations, such as wearable devices, metabolic analyzers, motion capture systems, and artificial intelligence, which enable high-resolution analysis of the body’s response to physical stress. As a result, exercise physiology has become an increasingly data-rich field, facilitating the design of personalized training approaches that account for genetic, physiological, and behavioral diversity among athletes.

Furthermore, the scope of exercise physiology continues to broaden, extending beyond athletic populations to include children, older adults, and individuals with chronic conditions [19,20,21]. This shift reflects a growing recognition that while the fundamental physiological mechanisms governing exercise adaptation are universal, they can be modified through targeted interventions. Importantly, insights gleaned from elite sports research now inform evidence-based guidelines for clinical rehabilitation, functional recovery, and public health initiatives, efforts aimed at addressing sedentary lifestyles and combating chronic diseases such as cardiovascular disease, obesity, and diabetes.

Building upon these developments, the second edition of the Special Issue “Focus on Exercise Physiology and Sports Performance” aims to capture these latest scientific and practical advancements. This collection seeks to advance our understanding of how the human body responds to and benefits from exercise, how these responses can be optimized for performance and health, and how interdisciplinary collaboration continues to expand the boundaries of human movement science.

## 2. An Overview of Published Articles

This second edition features a total of seventeen contributions that collectively reflect the breadth and complexity of current research in exercise physiology. A concise overview of the main themes and findings of these papers is presented below.

Chang et al. (contribution 1) investigated the comparative effects of absolute and incremental blood flow restriction training (BFRT) under matched cuff pressures on muscle strength and body composition in previously untrained adults. Over an eight-week intervention, both BFR protocols produced significant improvements in lower body maximal strength, comparable to those achieved through traditional high-load resistance training (HL-RT). Although HL-RT yielded greater increases in thigh circumference, changes in overall muscle mass and fat-free mass did not differ between the groups. These findings suggest that low-load BFRT, whether fixed or progressive, can effectively enhance strength with minimal mechanical stress, offering a practical alternative to conventional resistance programs.

In a meta-analysis by Chen et al. (contribution 2), the effects of aerobic exercise (AE) on blood lipid profiles in individuals with overweight or obesity were systematically evaluated. Drawing upon nineteen eligible studies, their findings revealed that AE significantly improved triglycerides, total cholesterol, and low-density lipoprotein (LDL) levels, while elevating high-density lipoprotein (HDL). Both moderate- and vigorous-intensity AE effectively reduced adverse lipid markers, although moderate-intensity exercise was uniquely associated with increased HDL. Moreover, continuous and interval AE yielded distinct benefits across lipid parameters, and longer intervention durations correlated with greater LDL reductions. These results reinforce AE as a key strategy for improving lipid metabolism and cardiovascular health in populations with overweight.

Deng et al. (contribution 3) conducted a comprehensive meta-analysis to examine the influence of altitude training on athletes’ aerobic capacity and identify the most effective training strategies. Synthesizing evidence from thirteen randomized controlled trials (RCTs), their analysis demonstrated that altitude exposure significantly enhanced hematological adaptations, including increases in hemoglobin concentration and total hemoglobin mass, thereby improving endurance-related performance outcomes. However, no substantial improvement was observed in maximal oxygen uptake. Subgroup findings indicated that the “live high, train high” model and interventions exceeding three weeks were particularly effective. These results emphasize the physiological benefits and practical considerations of altitude-based conditioning for endurance athletes.

Bai et al. (contribution 4) conducted a meta-analysis to determine the effects of eccentric training on upper limb muscle strength and identify optimal training parameters. Analyzing data from eleven studies, they confirmed that eccentric training significantly enhances upper limb strength, with the greatest improvements being observed after 4–8 weeks of intervention. High-intensity and rapid eccentric contractions elicited superior adaptations compared with moderate-intensity or slower movements. These findings highlight that time-efficient, high-intensity eccentric protocols, particularly those emphasizing fast contraction velocities, can serve as an effective strategy for developing upper limb strength in both athletic and rehabilitation settings.

Li et al. (contribution 5) examined how variations in body mass index (BMI) influence movement coordination and energy transfer during sit-to-stand transitions in young adults. Their study revealed that individuals with overweight relied more heavily on trunk and pelvic power to compensate for reduced hip drive, resulting in altered coordination patterns and increased joint loading. Conversely, underweight participants demonstrated greater lower limb flexibility and utilized trunk–pelvis coordination to maintain stability. These findings highlight the impact of BMI on neuromuscular strategies and suggest that targeted exercise interventions could optimize movement efficiency and reduce injury risk across different weight categories.

Tsai et al. (contribution 6) investigated the physiological, metabolic, and running dynamics differences between level and inclined treadmill protocols to evaluate their impact on training intensity determination. Their results indicated that while key physiological markers, such as VO_2_max and gas exchange thresholds, remained similar across protocols, metabolic responses and running kinematics—including running economy, stride length, and ground contact time—differed significantly at higher intensities. These findings suggest that intensity zones established on inclined treadmills may not accurately translate to flat-surface running. The study underscores the importance of protocol specificity when designing and prescribing endurance training programs for athletes.

Ramadan et al. (contribution 7) investigated the association between alpha-actinin-3 (ACTN3) and peroxisome proliferator-activated receptor-alpha (PPARα) gene polymorphisms and athletic performance in Egyptian adolescent athletes. Their findings revealed a markedly higher prevalence of the ACTN3 “R” allele and PPARα “C” allele among athletes than in sedentary controls. Similarly, the R/R and C/C genotypes were significantly more frequent in athletes, suggesting that these genetic variants may contribute to enhanced physical performance. These results underscore the potential role of specific genotypes in predicting athletic aptitude and highlight the importance of integrating genetic insights with training strategies to optimize performance in youth sport populations.

Cheng et al. (contribution 8) investigated the acute effects of rest redistribution (RR) versus traditional set (TS) resistance training on vertical jump performance, heart rate variability (HRV), and perceived exertion (RPE) in anxious female college students. Their results demonstrated that while both protocols similarly reduced squat and countermovement jump metrics, RR preserved autonomic function and significantly lowered perceived fatigue compared with TS. These findings suggest that RR can maintain performance outcomes while reducing physiological stress and subjective exertion. Consequently, RR may serve as a practical, low-stress resistance training strategy for anxiety-prone populations, supporting both mental well-being and physical performance.

Ren et al. (contribution 9) examined the dose–response effects of inspiratory muscle training (IMT) on respiratory function and exercise performance in amateur male runners. Their findings revealed that both high- and low-intensity IMT significantly enhanced maximal inspiratory and expiratory pressures, extended time to exhaustion, reduced blood lactate accumulation, and lowered perceived exertion and breathlessness during exercise. Notably, high-intensity IMT produced superior improvements in exercise tolerance. These results highlight IMT as an effective adjunct to conventional training for enhancing respiratory muscle strength and endurance, suggesting that targeted high-intensity protocols may be particularly beneficial for optimizing performance in recreational runners.

Liu et al. (contribution 10) conducted a systematic review and meta-analysis to compare the effects of hypoxic training (HT) and normoxic training (NT) on cardiometabolic health in adults with overweight and obesity. Their analysis of 17 RCTs revealed that HT significantly enhanced cardiorespiratory fitness (CRF) compared with NT, while improvements in systolic and diastolic blood pressure were similar between the two conditions. Subgroup analyses indicated that younger participants (<45 years), shorter sessions (<45 min), higher exercise intensities, and FiO_2_ > 15% were particularly responsive to HT. These findings suggest HT as a promising strategy to improve CRF in this population.

Ma et al. (contribution 11) systematically investigated the effects of cold-water immersion (CWI) alone versus CWI combined with other recovery modalities (CWI + Other) on post-exercise fatigue. Their meta-analysis of 24 studies including 475 participants showed that both interventions effectively reduced delayed-onset muscle soreness (DOMS), with CWI + Other yielding a greater effect, particularly in athletes. Additionally, CWI + Other significantly lowered post-exercise C-reactive protein (CRP), suggesting anti-inflammatory benefits. No substantial differences were observed in creatine kinase levels or countermovement jump performance. These findings highlight that combining CWI with other therapies may enhance recovery and reduce exercise-induced inflammation more effectively than CWI alone.

Korivi et al. (contribution 12) reviewed the role of exercise training in mitigating chronic metabolic diseases among older adults. Their synthesis highlighted how aerobic, resistance, and combined training improve cardiopulmonary function, including oxygen consumption, pulmonary ventilation, and blood gas regulation, which are critical for meeting increased metabolic demands during activity. The review also emphasized that factors such as age, BMI, lifestyle behaviors, and comorbidities influence these physiological responses. By underscoring exercise as an effective non-pharmacological strategy, the study supports the promotion of regular physical activity to enhance respiratory efficiency and prevent or manage obesity, diabetes, cardiovascular disease, and related conditions in aging populations.

Chen et al. (contribution 13) systematically evaluated the impact of integrated neuromuscular training (INT) on athletic performance across 19 RCTs involving 783 young athletes. Their meta-analysis revealed that INT significantly enhanced jump, sprint, balance, and agility outcomes, with female athletes demonstrating greater gains in sprint and balance than males. Notably, interventions that were shorter than eight weeks, with sessions under 30 min and frequencies exceeding three times per week, were associated with more pronounced improvements. These findings underscore the value of INT as a multidimensional training approach, highlighting the importance of targeted program design to optimize specific physical performance indicators in athletic populations.

Chen et al. (contribution 14) conducted a meta-analysis to evaluate the effects of BFRT on glucose and lipid metabolism in adults with overweight and obesity. Their analysis of eight RCTs involving 267 participants revealed that BFRT significantly improved fasting blood glucose and insulin resistance (HOMA-IR), highlighting its potential for enhancing glycemic control. However, no meaningful changes were observed in lipid profiles, suggesting a limited impact on lipid metabolism. These findings support BFRT as a time-efficient intervention for improving glucose regulation in adults with overweight or obesity, while underscoring the need for further research to clarify its effects on lipid outcomes.

Meng et al. (contribution 15) systematically reviewed the effects of inorganic nitrate supplementation on muscle performance in healthy females during high-intensity, short-duration exercise. Their meta-analysis of RCTs revealed that nitrate intake produced a small-to-moderate improvement in muscular power, whereas the effects on muscle strength and sprint performance were negligible. These findings underscore the importance of sex-specific investigations in sports nutrition, as most prior research has focused on male participants. Practically, targeted nitrate supplementation may offer modest benefits for power output in female athletes, highlighting the need for individualized strategies to optimize training adaptations and performance outcomes.

Shi et al. (contribution 16) investigated how different foot strike patterns (forefoot, midfoot, and rearfoot) affect lower limb muscle activation in healthy male university students. Using EMG and time–frequency analyses, they found that rearfoot strike induced the earliest peak muscle frequency during mid-stance, while forefoot strike exhibited higher high-frequency energy in the medial gastrocnemius. Moreover, co-activation of ankle dorsiflexors and plantar flexors was greater in midfoot and rearfoot strikes compared with forefoot. These findings provide important insights into neuromuscular control during running and suggest that foot strike patterns can influence muscle pre-activation and load distribution, which may inform injury prevention strategies for recreational runners.

Liu et al. (contribution 17) examined sex-specific differences in foot arch structure and function and their impact on postural control and energy flow during the sit-to-stand task. Their results indicated that males exhibited greater arch stiffness, lower mediolateral center of pressure (COP) frequency, and higher segmental net power in the thigh and shank, whereas females relied on more frequent postural adjustments and showed distinct patterns of joint power. These differences suggest that reduced arch stiffness in females may increase mechanical loading on the knee and ankle, potentially elevating injury risks. The findings highlight the importance of considering sex-specific biomechanics in training and injury prevention programs.

## 3. Conclusions

This Special Issue showcases the notable diversity and translational value of cutting-edge research in exercise science. Bringing together seventeen distinct contributions, it underscores the multifaceted impacts of exercise interventions, spanning physiological, metabolic, neuromuscular, and biomechanical outcomes, across a broad spectrum of populations. These groups range from sedentary adults with no prior training experience to elite athletes competing at the highest levels, as well as older adults living with chronic conditions.

A set of core themes emerges across the collection, including the effectiveness of innovative training methodologies. These include BFRT, INT, IMT, and specialized eccentric exercise protocols. Complementing these intervention-focused studies is a strong emphasis on individualized factors that shape an individual’s response to exercise: biological sex, BMI, genetic predispositions that influence adaptation potential, and foot biomechanical characteristics that impact movement efficiency and injury risk.

Notably, several studies also address critical strategies for maximizing training benefits, such as evidence-based recovery approaches, targeted nutritional supplementation regimens, and environmental modifications (e.g., hypoxia exposure, altitude training). These factors are shown to play key roles in optimizing physiological adaptation and enhancing performance outcomes. Collectively, these findings reinforce a central tenet of modern exercise science: exercise functions simultaneously as a powerful tool for performance enhancement and a non-pharmacological intervention to promote health.

Overall, the collection captures the ongoing evolution of exercise physiology as a field: moving toward more personalized, interdisciplinary approaches that not only maximize human performance across diverse populations but also support long-term health and functional resilience.
